# Prevention of type 2 diabetes in a primary healthcare setting: Three-year results of lifestyle intervention in Japanese subjects with impaired glucose tolerance

**DOI:** 10.1186/1471-2458-11-40

**Published:** 2011-01-17

**Authors:** Naoki Sakane, Juichi Sato, Kazuyo Tsushita, Satoru Tsujii, Kazuhiko Kotani, Kokoro Tsuzaki, Makoto Tominaga, Shoji Kawazu, Yuzo Sato, Takeshi Usui, Isao Kamae, Toshihide Yoshida, Yutaka Kiyohara, Shigeaki Sato, Hideshi Kuzuya

**Affiliations:** 1 Division of Preventive Medicine, Clinical Research Institute, National Hospital Organization Kyoto Medical Center, Kyoto, Japan; 2 Department of General Medicine, Nagoya University Hospital, Nagoya, Japan; 3 Comprehensive Health Science Center, Aichi Health Promotion Foundation, Aichi, Japan; 4 Diabetes Center, Tenri Yorozu-sodansho Hospital, Tenri, Japan; 5 Department of Clinical Laboratory Medicine, Jichi Medical School, Tochigi, Japan; 6 Division of Internal Medicine, Hananoki Hospital, Tochigi, Japan; 7 Department of Diabetes and Metabolism,Marunouchi Hospital, The Institute for Adult Diseases, Asahi Life Foundation, Tokyo, Japan; 8 Department of Health Science, Faculty of Psychological and Physical Science, Aichi Gakuin University, Aichi, Japan; 9 Division of Endocrinology, Clinical Research Institute, National Hospital Organization Kyoto Medical Center, Kyoto, Japan; 10 JPMA Pharmacoeconomics Program, Graduate School of Health Management, Keio University, Fujisawa, Japan; 11 Department of Diabetes and Metabolism, Kyoto City Hospital, Kyoto, Japan; 12 Department of Medicine and Clinical Science, Graduate School of Medical Sciences, Kyusyu University, Fukuoka, Japan; 13 Aino Hospital, Ibaraki, Japan; 14 National Hospital Organization Kyoto Medical Center, Kyoto, Japan; 15 Higashiyama Takeda Hospital, Kyoto, Japan

## Abstract

**Background:**

A randomized control trial was performed to test whether a lifestyle intervention program, carried out in a primary healthcare setting using existing resources, can reduce the incidence of type 2 diabetes in Japanese with impaired glucose tolerance (IGT). The results of 3 years' intervention are summarized.

**Methods:**

Through health checkups in communities and workplaces, 304 middle-aged IGT subjects with a mean body mass index (BMI) of 24.5 kg/m^2 ^were recruited and randomized to the intervention group or control group. The lifestyle intervention was carried out for 3 years by public health nurses using the curriculum and educational materials provided by the study group.

**Results:**

After 1 year, the intervention had significantly improved body weight (-1.5 ± 0.7 vs. -0.7 ± 2.5 kg in the control; p = 0.023) and daily non-exercise leisure time energy expenditure (25 ± 113 vs. -3 ± 98 kcal; p = 0.045). Insulin sensitivity assessed by the Matsuda index was improved by the intervention during the 3 years. The 3-year cumulative incidence tended to be lower in the intervention group (14.8% vs.8.2%, log-rank test: p = 0.097). In a sub-analysis for the subjects with a BMI > 22.5 kg/m^2^, a significant reduction in the cumulative incidence was found (p = 0.027).

**Conclusions:**

The present lifestyle intervention program using existing healthcare resources is beneficial in preventing diabetes in Japanese with IGT. This has important implications for primary healthcare-based diabetes prevention.

**Trial registration number:**

UMIN000003136

## Background

The incidence of type 2 diabetes is increasing in Japan [1]. Although Japanese have a lower prevalence of obesity than Westerners, a tendency to gain weight due to lifestyle changes coupled with an aging of the population seems to be closely related to the rapid expansion of the diabetic population [1]. There is thus an urgent need for effective public health strategies to combat this situation in Japan.

There is now substantial evidence that the development of type 2 diabetes can be prevented or delayed in high-risk subjects through lifestyle intervention [2-8]. The Finnish Diabetes Prevention Study (DPS) [4] and the US Diabetes Prevention Program (DPP) [5] have clearly shown that, in obese subjects with impaired glucose tolerance (IGT), lifestyle changes associated with a 5-7% decrease in body weight resulted in a 58% reduction in the development of diabetes. Thus lifestyle modifications are considered the most effective means of delaying or preventing the development of type 2 diabetes. There are several examples in the literature about the various levels of effectiveness of lifestyle intervention [9]. In both the DPP [5] and DPS [4], considerable efforts were made by well-trained staff to achieve changes in lifestyle among participants. However, results are not consistent across studies in primary healthcare settings. How to translate the findings of clinical research, such as the DPS and DPP, into real-world practice [10,11] is a key issue to be addressed. In Japan, by law, much of the adult population undergoes a health checkup every year in the workplace or at community centers. The checkups have revealed a huge number of subjects at a high risk for developing type 2 diabetes. These people are usually given simple information and guidance about diabetes and a healthy lifestyle. Despite this approach, the diabetic population has increased at the national level, probably due to a lack of evidence-based methodologies of lifestyle intervention and mechanisms to implement these widely at public health care levels. It is not known to what extent lifestyle intervention in a primary healthcare setting is effective. The present study is a randomized control trial to test the feasibility and effectiveness of a lifestyle intervention program, carried out in a primary healthcare setting using existing resources, in Japanese with IGT. We found that this relatively modest intervention could produce beneficial effects on the incidence of type 2 diabetes over a 3-year period. This has important implications for primary healthcare-based diabetes prevention.

## Methods

The study protocol was approved by the Ethics Committee of the National Hospital Organization Kyoto Medical Center, and all subjects gave their written informed consent before the start of the study. Thirty-two community health care institutions and company clinics across the country participated in the study as collaborative centers. In each center, a public health nurse was appointed as a study nurse for recruitment, intervention, laboratory referral, and clinical measurements.

### Study design and subjects

**Figure 1:**
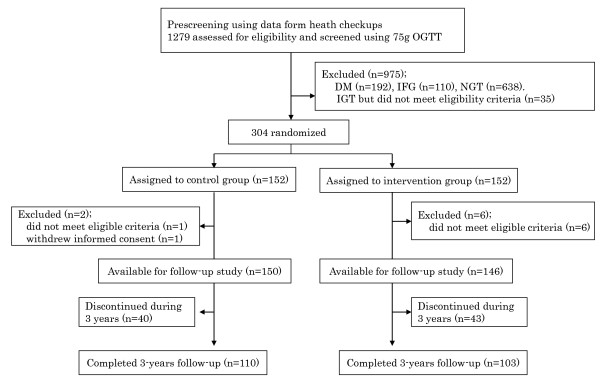
**Recruitment, random assignment, and 3 - year follow-up of study subjects**. DM: diabetes mellitus, IFG: impaired fasting glucose, NGT: normal glucose tolerance

Subjects with IGT, aged 30-60 years, were recruited through health checkups conducted at each collaborative center. The recruitment started in March 1999 and was completed in December 2002. A two-step strategy was adopted for identifying subjects with IGT as described previously [12]. Using the data from health checkups, those who met one of the following criteria were extracted: 1) fasting plasma glucose (FPG) concentration ≥5.6 mmol/l but < 7.0 mmol/l, 2) casual plasma glucose (CPG) concentration ≥7.8 mmol/l but <11.1 mmol/l when blood is drawn within 2 hours after a meal, or CPG concentration ≥6.1 mmol/l but <7.8 mmol/l when blood is drawn 2 hours or more after a meal, or 3) IGT as indicated by a previous 75 g oral glucose tolerance test (OGTT). Those with 1) a previous diagnosis of diabetes mellitus other than gestational diabetes, 2) a history of gastrectomy, 3) physical conditions such as ischemic heart disease, heart failure, exercise-induced asthma, and orthopedic problems where exercise was not allowed by a doctor, 4) definitive liver and kidney diseases, 5) autoimmune diseases, and 6) a habit of drinking heavily (69 g or more of ethanol per day) [13] were excluded. Those who had already begun lifestyle modifications, such as routine moderate to vigorous exercise, were also excluded. Thus it should be noted that the findings obtained cannot be generalized to all high-risk people with IGT. It was roughly estimated that there were more than 10,000 people with borderline hyperglycemia at the 32 collaborative centers. Each center recruited study candidates using posters, through fliers, and by word of mouth. Figure 1 shows a flow diagram for recruiting study subjects. Altogether, 1279 subjects who met the criteria and gave written informed consent, underwent a 75 g OGTT. Diabetes and IGT were diagnosed based on the World Health Organization (WHO)'s criteria [14].

Finally, 304 subjects diagnosed with IGT were randomly assigned to either a lifestyle intervention group or a control group by the committee of the study group. Two subjects from the control group and 6 from the intervention group were excluded from the study, since it turned out that they did not meet the eligibility criteria. The result of the randomization was unmasked to the participants, those administering the interventions, and those assessing the data. The average number of participants per center (including both the control and intervention groups) was 9. We planned to follow-up the participants for 6 years regarding the development of diabetes.

According to prospective studies on the Japanese population, the yearly incidence of diabetes among subjects with IGT varies between 1 and 5% [15-17]. Therefore, it was assumed that the 6-year cumulative incidence of diabetes would be 30% in the control group. The present study was designed to detect a 50% reduction in the incidence by the intervention. Thus the sample size required was 313 with a type 1 error of 5%, with 80% power (beta = 20%) at the two-tailed 5% significance level, and allowing for a withdrawal rate of 30%.

### Intervention

The follow-up of the participants started in April 1999 and the last case completed a three-year follow-up in January 2006.

The goals of intervention were: 1) to reduce initial body weight by 5% in overweight and obese subjects, and 2) to increase energy expenditure due to leisure time physical activity (LTPA) by 700 kcal per week. The interventions were carried out by the study nurse in each collaborative center in the form of both group and individual sessions, using the guideline, curriculum, and educational materials provided by the committee of the study group. When needed, the study nurse could ask a part-time dietician for diet counseling. A 27-page booklet titled "Change Your Lifestyle to Prevent Diabetes" was given to each participant as a guide. During the initial six months, four group sessions were conducted using slides, videotapes, and a booklet with each session lasting two or three hours. The main subjects in each group session were as follows: (1) What is diabetes?, What is IGT?, How to prevent diabetes?, (2) Healthy diets to prevent diabetes, (3) Exercise tips to prevent sporting injuries, and (4) Let's enjoy exercise. The individual session was conducted biannually during the three years with each session lasting 20 to 40 minutes. Personalized goals, such as a minimum of 20 minutes' moderate walking each day, were set. The session was conducted based on theoretical concepts and techniques for behavioral change, such as self-efficacy, self-monitoring, and the transtheoretical model [18]. After the first year, contact by telephone could replace the individual face to face sessions. The study subjects attended both group and individual sessions by themselves without any support person.

An assessment of the dietary intake of each participant was conducted using a semiquantitative food frequency questionnaire (FFQ) [19] with photographs of 122 varieties of dishes and foods. Each item was shown with a real portion size. The subjects were advised to take the proper amount of calories, decrease the mean percent of energy derived from dietary fat to less than 25%, and restrict daily alcohol consumption to less than 160 kcal. They were also advised to eat three meals a day and avoid eating late at night. Self-reported levels of LTPA were assessed using a physical activities questionnaire [20]. To achieve the exercise goal, aerobic exercise such as walking was recommended. Data on dietary intake and physical activities were assessed by the study group and the results were sent back to study nurses at each collaborative center.

To reinforce the intervention, between-visit contact by fax was also made monthly during the initial twelve months. Simple cartoons were drawn on the fax sheet to give tips for improving lifestyle.

The control group received only one group session on a healthy lifestyle and the prevention of diabetes at the baseline. No individual guidance was given during the study period. However, the control group received anthropometric and blood examinations regularly during the study as did the intervention group.

### Measurements

Anthropometric (height, body weight, and waist circumference) and blood pressure measurements were done every three months during the first year and biannually thereafter. Waist circumference was measured at the umbilical level. Biochemical studies, including a 75 g-OGTT, were conducted biannually during the first year and annually thereafter. Total cholesterol, high-density lipoprotein (HDL)-cholesterol, triglyceride, creatinine, uric acid, aspartate aminotransferase (AST), alanine aminotransferase (ALT), gamma-glutamyltransferase (GGT), HbA1c, plasma glucose, and insulin levels were measured at a central laboratory (SRL Co. Ltd.,, Tokyo, Japan). For the intervention group, the results of these measurements were given back individually to each subject in the intervention group during individual sessions with the study nurse. For the control group, the results were sent by mail with brief comments. The assessment of dietary intake was conducted annually. Levels of LTPA were assessed biannually during the first year and annually thereafter. Pancreatic β cell function and insulin resistance were assessed using the homeostasis model assessment (HOMA-β and HOMA-IR, respectively) [21]. An insulin sensitivity index (Matsuda index) was also calculated using insulin and glucose data obtained from 75 g OGTTs [22,23]. Body mass Index (BMI) was calculated as weight in kilograms divided by height in meters squared. "Overweight" and "obese" were defined according to the WHO recommendations for Asians [24]. All clinical and diet and exercise data were collected at each collaborative center by the study nurse and sent to the study group for analysis.

### Endpoint

The primary endpoint was the development of diabetes, diagnosed and confirmed by two consecutive 75 g-OGTTs. The diagnosis of diabetes was based on the WHO's criteria [14].

### Training of the study nurses

The study group organized a one and a half day study meeting for the study nurses in the beginning and annually thereafter. The meeting was designed to 1) standardize the intervention method, 2) improve their skills for eliciting motivation from the participants to achieve the lifestyle goals, and 3) increase their knowledge on diabetes, nutrition, exercise, and behavioral modification. The attendance rate for the nurses was almost 100% in the initial training course and between 70 and 90% for the annual training course after 1 year.

### Statistical analysis

All data are presented as the means ± SD. Comparisons of baseline values and mean changes from baseline to year 1 between the groups were made with a two-tailed unpaired t test or the χ^2 ^test when applicable. A two-tailed paired t-test was used to analyze differences within groups between the baseline and year 1. Survival curves were calculated to estimate the cumulative incidence of diabetes. The difference between the groups in the incidence of diabetes was tested by means of the two-sided log-rank test. A p value less than 0.05 was considered statistically significant. The analyses were done using the SPSS/PC statistical program (version 11.1 for windows; SPSS, Inc., Chicago, IL, USA).

## Results

We randomly assigned the 304 subjects with IGT to two groups and analyzed the data for 296 individuals (150 in the control group and 146 in the intervention group) (Figure 1). A total of 83 subjects (28%) withdrew from the study before the 3-year mark (40 in the control group and 43 in the intervention group). The withdrawals were due to personal reasons (moving etc) in 18 cases, medical reasons in 5, and loss of contact in 40. Twenty subjects were not able to continue the study for reasons related to the collaborative centers themselves, such as the closure of a center. The rate of withdrawal was higher among men than women (36.9% vs. 19.0%, p < 0.01). No differences were found in age and BMI between those who withdrew from the study before the 3-year mark and those who continued. The baseline characteristics of both the control and intervention groups were similar as regard to age (51 ± 6 and 51 ± 7, respectively) and male to female ratio (76/74 and 74/74, respectively), and proportion of overweight (23.0≤BMI<27.4: 48.5% and 50.0%, respectively) and obese (BMI ≥27.4: 18.6% and 18.8%, respectively) people. There was no difference in exercise LTPA between the groups at the baseline (p = 0.197), although non-exercise LTPA (below 3 METs) was significantly greater in the control group (p = 0.043). Non-exercise LTPA included gardening, shopping, Sunday carpentering, playing musical instruments, and so on. There were no significant differences in other lifestyle, anthropometric, and biochemical measurements at the baseline between the groups (Table 1). Thus we were able to successfully assign the cohort of subjects to two groups.

**Table 1:** Baseline and 1-year or 3-year follow-up data in the control and intervention groups.

Parameters	Control group			Intervention group			*P value *^*b*^	
	
	Baseline ^a ^ (n = 131)	1-year (n = 131)	3-year (n = 110)	Baseline (n = 123)	1-year (n = 123)	3-year (n = 103)	at 1-year mark	at 3-year mark
Energy intake (kcal)	2455 ± 838	2292 ± 739*	2153 ± 734*	2299 ± 788	2097 ± 895*	2016 ± 677*	0.647	0.794
Fat ^c ^(%)	27.5 ± 5.2	27.4 ± 5.2	27.8 ± 5.4	26.5 ± 5.6	25.5 ± 5.6*	25.7 ± 5.2	0.088	0.110
Alcohol (g)	21.0 ± 36.1	18.6 ± 29.2	13.7 ± 23.2*	20.1 ± 44.8	24.6 ± 87.7	15.7 ± 29.8	0.171	0.149
Leisure time physical activity (kcal)	136 ± 159	163 ± 172*	181 ± 201*	91 ± 132	155 ± 180*	161 ± 215*	0.078	0.214
Exercise (kcal)	57 ± 79	86 ± 99*	92 ± 105*	43 ± 88	82 ± 122*	74 ± 117*	0.474	0.958
Exercise (minutes per week)	118 ± 160	184 ± 206*	185 ± 229*	91 ± 187	184 ± 262*	160 ± 229*	0.339	0.556
Non-exercise (kcal) ^d^	79 ± 139	76 ± 133	90 ± 174	49 ± 85	74 ± 119*	88 ± 186*	0.045	0.148
Weight (kg)	63.9 ± 11.7	63.1 ± 11.7*	62.5 ± 11.2*	64.9 ± 12.9	63.5 ± 12.9*	63.1 ± 12.9*	0.023	0.069
Body mass index (kg/m^2^)	24.5 ± 3.2	24.2 ± 3.1*	24.4 ± 3.3*	24.8 ± 3.6	24.2 ± 3.6*	24.3 ± 3.7*	0.022	0.051
Waist circumference (cm)	84.4 ± 9.4	83.3 ± 8.6*	84.2 ± 9.5	85.9 ± 10.9	84.2 ± 10.5*	84.7 ± 11.9	0.309	0.362
Fasting plasma glucose (mmol/l)	6.1 ± 0.5	5.9 ± 0.6	6.0 ± 0.9	5.9 ± 0.5	5.8 ± 0.6*	6.0 ± 0.8	0.698	0.481
2-hplasma glucose (mmol/l)	9.0 ± 0.9	8.3 ± 2.0*	8.5 ± 2.4	9.2 ± 0.9	8.0 ± 2.1*	8.4 ± 2.5*	0.083	0.553
Fasting insulin (pmol/l)	43.8 ± 21.6	44.4 ± 40.8	45.8 ± 23.9	43.2 ± 22.2	44.4 ± 25.2	47.6 ± 36.1	0.861	0.632
2-h insulin (pmol/l)	330.6 ± 211.8	308.4 ± 178.8	377.4 ± 280.7	337.8 ± 199.8	342.0 ± 271.2	390.0 ± 374.2	0.413	0.999
Matsuda index ^e^	5.4 ± 3.5	5.6 ± 3.3	5.3 ± 3.2	4.8 ± 2.3	5.9 ± 3.7*	5.5 ± 3.4*	<0.001	<0.001
Aspartate aminotransferase (IU/l)	25 ± 8	25 ± 12	26 ± 15	25 ± 12	23 ± 13	25 ± 17	0.170	0.977
Alanine aminotranseferase (IU/l)	25 ± 16	26 ± 17	27 ± 16	26 ± 18	24 ± 17	25 ± 14	0.212	0.520
Gamma-glutamyltransferase (IU/l)	53 ± 58	59 ± 91	59 ± 97*	48 ± 46	44 ± 47*	43 ± 66	0.041	0.158

Data are means ± SDs. ^a ^There were no significant differences in any of the baseline variables between the control and intervention groups except for non-exercise physical activity. ^b ^P values for differences in change between groups. ^c ^Proportion of energy derived from dietary fat. ^d ^Non-exercise leisure time physical activity includes gardening, carpentry, shopping, and playing a musical instrument. ^e ^The Matsuda index is an insulin sensitivity index derived from oral glucose testing. * P value < 0.05 (Baseline vs. 1-year or 3-year).

Table 1 shows mean changes in lifestyle, anthropometric, and biochemical parameters from the baseline at the 1-year and 3-year marks in both groups. In the intervention group, the mean daily energy intake decreased by 202 kcal and mean daily energy expenditure by LTPA increased by 64 kcal at the 1-year mark. These beneficial lifestyle changes were observed even at the 3-year mark. Body weight, BMI, waist circumference, and systolic and diastolic blood pressure (not shown in the Table 1) decreased significantly from the baseline at the 1-year mark. The changes in body weight and BMI were seen also at the 3-year mark.

Although fasting and 2 hour plasma glucose decreased, fasting and 2 hour insulin concentrations did not change during the three years. HOMA-IR and HOMA-β did not change either (data not shown). However, Matsuda index, as a marker of whole body insulin sensitivity calculated using plasma glucose and serum insulin levels from 75 g OGTTs, increased from the baseline at both the 1-year and 3-year marks. Serum GGT levels decreased at the 1-year mark. Serum HDL cholesterol levels increased at the 1-and 3-year marks while serum triglyceride and cholesterol levels did not change (data not shown). Beneficial changes were also found in the control group although to a lesser extent. Between the groups, changes in daily energy expenditure due to non-exercise LTPA, body weight and BMI, serum GGT levels, and the Matsuda index were significantly different at the 1-year mark. These differences were not significant at the 3-year mark except for the Matsuda index. The difference in the Matsuda index remained significant even at the 3-year mark.

**Figure 2:**
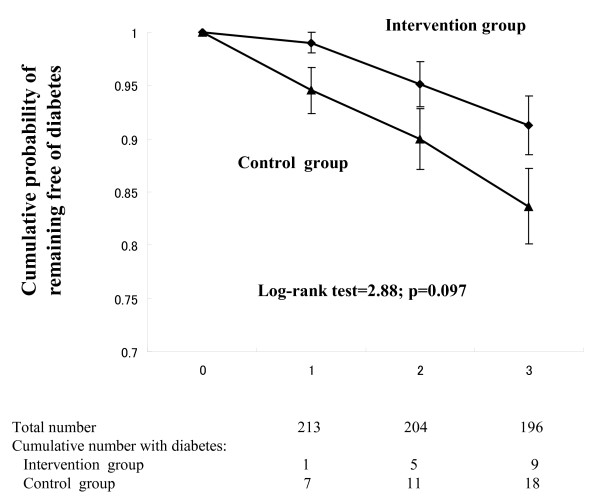
**Cumulative incidence of diabetes over the three years in all participants. **Kaplan-Meier plots. Intervention group (black circle) and Control group (black triangle).

Diabetes was diagnosed in a total of 27 subjects during the three years; 9 in the intervention group and 18 in the control group. The estimated cumulative incidence of diabetes over the 3-year period was 8.2% in the former and 14.8% in the latter. The relative risk reduction was thus 53% with the intervention [95% confidence interval (CI); 0.25-1.13]. The difference between the groups, however, did not reach a level of statistical significance (log-rank test: p = 0.097) at the 3-year mark (Figure 2). Our study group included both lean and obese subjects with a BMI ranging widely from 16.8 to 39.6 kg/m^2^. It may be thus possible that the heterogeneity in BMI in our cohort accounts for the statistically insignificant results. To examine if the effects of lifestyle intervention alter with BMI, the participants were then stratified into quartiles according to the baseline BMI. Diabetes developed in 5 out of 52 in the lowest quartile (2 from the control group and 3 from the intervention group) during the 3 years. Thus the effect of lifestyle intervention was not apparent in this lowest BMI quartile. The sub-analysis for the subjects with a BMI>22.5, however, revealed a significant decrease in the cumulative incidence with the intervention (log-rank test: p = 0.027). There was no difference in changes in BMI, waist circumference, and serum lipid levels between the lowest BMI quartile and the upper BMI quartiles in the intervention group. The change in serum ALT was significantly improved in the upper 3 BMI quartiles than the lowest BMI quartile at the 1-year mark (-3 ± 16 IU/l vs. +3.0 ± 9 IU/l; p = 0.010), although there was no difference in the control group (+1 ± 14 IU/l vs. 0 ± 15 IU/l; p = 0.498). The Matsuda index of the upper 3 BMI quartiles in the intervention group was significantly improved than in the control group at the 1-year mark (+1.1 ± 3.0 vs. -0.2 ± 3.6; p = 0.026), although there was no difference in the lowest BMI quartile between groups (+1.3 ± 3.4 vs. +1.0 ± 3.7; p = 0.702).

## Discussion

This is the first randomized control trial to test whether a lifestyle intervention, carried out on a community or workplace basis using existing healthcare resources, can prevent or delay the development of type 2 diabetes in middle-aged Japanese with IGT.

The participants were recruited through health checkups at community health centers and in the workplace. They were all volunteers, who participated in response to posters, fliers, and word of mouth. Therefore it was likely that they were motivated and prepared to alter their lifestyle, at least in the beginning. The rate of withdrawal before the 3-year follow-up was, however, high (28%). About one third of male participants withdrew from the study. This might represent the limitations of intervention carried out in a primary healthcare setting. Generally speaking, middle-aged men in Japan tend to prioritize work over health. Therefore, modifying lifestyle among the middle-aged was a challenge.

Compared with the DPS [4] and DPP [5], the present study had a less intensive intervention. The majority of the public health nurses, reflecting the real world primary healthcare setting, did not have special training in lifestyle modifications. At a feasible level, they carried out the intervention using the protocol and educational materials provided by the study group. As a rule, the same study nurse carried out the interventions on the same participant during the study. But this was not always possible due to a personnel change at the collaborative center.

We found improvements in lifestyle and anthropological and biochemical parameters with the intervention. However, between the intervention and control groups, differences in changes from the baseline were statistically significant only in increases in energy expenditure due to non-exercise LTPA, in weight reduction and, among biochemical parameters, in serum GGT levels and the Matsuda index. The mean body weight reduction was very modest, being 1.5 ± 2.7 kg (2.3%) in the intervention group and 0.7 ± 2.5 kg (1.3%) in the control group at the 1-year mark. At the 3-year mark, the differences between the groups were not statistically significant for any of the parameters except the Matsuda index. Thus it was suggested that the improvement in insulin sensitivity assessed by the Matsuda index was maintained during the three years.

In this study, four group sessions were given to the intervention group during the initial 6 months, while one session was given to the control group about diabetes mellitus and a healthy lifestyle at the baseline. The control group, however, underwent physical and blood examinations regularly during the study as did the intervention group. In addition, as the study subjects were individually randomized at each collaborating center, exchanges of information among participants at the same collaborative centers could have happened. All these factors might lead to difficulties in obtaining statistically significant differences between the groups. Therefore, it would be more appropriate to refer to the groups as a conventional intervention group and an intensive intervention group instead of a control group and an intervention group, respectively.

Most importantly, we found that this relatively modest intervention could produce beneficial effects on the incidence of type 2 diabetes during a 3-year period. The halving (51%) of the relative risk for overall subjects through this intervention is not negligible, even though it did not reach a statistically significant level. Our cohort was heterogeneous in BMI with 30% of the subjects having a normal or lower than normal BMI. Due to the small number of subjects in the present study, a subgroup analysis was difficult. But we found a significant reduction in cumulative incidence (log-rank test: p = 0.027) for the subjects with a BMI> 22.5. Thus the effects of the intervention for lean subjects might attenuate the impact on the incidence. Regarding this, it would be important to clarify an effective measure for the prevention of diabetes in subjects with a low BMI in future studies, since the Japanese IGT population includes a considerable number of such subjects.

In the DPP, weight reduction was found to be essential for the lifestyle intervention to be beneficial [5]. In an Indian Study [7], however, the benefits seemed independent of weight change. In a hospital-based lifestyle intervention, Kosaka concluded that the benefits of lifestyle intervention could not be solely ascribed to weight reduction [6]. The present study found that minimal weight reduction in the intervention group (less than 3% on average) lowered the relative risk to 53% over 3 years, similar to the risk reduction seen in the DPS and DPP (58%) where the subjects lost 5-7% of body weight on average. Thus it seems that the relationship between body weight and diabetes risk in Asians is not as straightforward as in Western people. Asians have lower BMI but higher body fat levels than do whites [25,26]. Japanese Americans are prone to develop visceral obesity and metabolic syndrome [27,28]. A reasonable explanation for the present findings might be a more profound reduction in specific fat depots, such as visceral fat and liver fat. It has been reported that lifestyle intervention with diet and physical activity is effective at reducing hepatic steatosis in patients with non-alcoholic fatty liver disease [29]. Although there was no difference in daily alcohol consumption between the groups, we found that serum GGT levels decreased in the intervention group, but increased in the control group. These findings are important, since it has been reported that the serum concentrations of GGT and ALT are a predictive marker of type 2 diabetes [30-33], even at concentrations still considered to be within the normal range [34]. Thus, the difference in the changes in GGT levels between the groups is likely to reflect changes in liver fat contents. Further examination including abdominal ultrasonography and computed tomography [35] will be needed.

## Conclusions

In conclusion, the present study suggests that lifestyle intervention using existing healthcare resources in communities and workplaces is beneficial in preventing or delaying the development of diabetes in middle aged Japanese with IGT. General improvements in lifestyle including dietary and exercise habits might be meaningful even if the weight reductions achieved are only modest. The findings have important implications for primary healthcare-based diabetes prevention.

## List of abbreviations used

ALT: Alanine aminotransferase; AST: Aspartate aminotransferase; BMI: Body mass index; CPG: Casual plasma glucose; DPP: Diabetes Prevention Program; DPS: Diabetes Prevention Study; FFQ: Food frequency questionnaire; FPG: Fasting plasma glucose; GGT: Gamma-glutamyltransferase; HDL: High-density lipoprotein; HOMA: Homeostasis model assessment; IGT: Impaired glucose tolerance; LTPA: Leisure time physical activity; OGTT: Oral glucose tolerance test.

## Competing interests

The authors declare that they have no competing interests.

## Authors' contributions

HK, the project leader, is involved in all aspects of the study. JS, ST, MT, SK, YS, IK, KY, and SS designed the study, and prepared the protocol of intervention. TK contributed to study design and coordination. NS, KK, and KT performed the statistical analysis and prepared the manuscript. TU and YT helped to draft the manuscript participated in the critical revision of the manuscript and the trial management. All authors have read and approved the final version of the manuscript.

## Pre-publication history

The pre-publication history for this paper can be accessed here:

http://www.biomedcentral.com/1471-2458/11/40/prepub
